# Artificial Intelligence-Based Adaptive Simulation Integrated With a Workforce Decision-Support System to Improve Simulated Nursing Leadership Performance: A Randomized Controlled Study

**DOI:** 10.7759/cureus.117315

**Published:** 2026-10-01

**Authors:** Mohammed Almalki

**Affiliations:** 1 Department of Nursing Administration and Education, Taif University, Taif, SAU

**Keywords:** adaptive simulation, artificial intelligence, decision-support systems, nursing leadership, simulation-based education

## Abstract

Background

The growing integration of artificial intelligence (AI) in healthcare necessitates that nurse leaders develop advanced competencies in decision-making, communication, and data-informed clinical judgment. Traditional simulation-based education, while effective, often relies on fixed scenarios with limited adaptability and minimal real-time decision support. AI-enhanced simulation offers a potential solution by creating responsive, adaptive learning environments that support leadership development.

Methods

This randomized controlled, parallel-group, pretest-posttest trial included 130 final-year nursing students who were randomly allocated to an intervention group (n = 65), receiving AI-adaptive simulation integrated with an AI-based workforce decision-support system, or a control group (n = 65). Twenty-one clinical preceptors served as trained and blinded outcome assessors and were not included in the randomized sample. Leadership performance was assessed using the Creighton Simulation Evaluation Instrument (C-SEI). Secondary outcomes included team performance, general self-efficacy, and AI acceptance.

Results

At Week 10, the intervention group demonstrated greater improvement in leadership performance than the control group (between-group difference in change = 5.20, 95% CI 4.18-6.22, p < 0.001), with a large standardized effect (Cohen's d = 1.78). Greater improvements were also observed in team performance (between-group difference in change = 0.63, 95% CI 0.44-0.82, p < 0.001, d = 1.24) and general self-efficacy (between-group difference in change = 5.30, 95% CI 3.82-6.78, p < 0.001, d = 1.37). Intervention-group students reported favorable AI acceptance, including perceived usefulness and ease of use.

Conclusions

AI-adaptive simulation integrated with an AI-based workforce decision-support system was associated with improved leadership performance, team performance, and self-efficacy among final-year nursing students under controlled simulation conditions. Further research is needed to determine whether these effects are sustained and transfer to clinical practice.

## Introduction

Healthcare environments are becoming increasingly data-intensive, fast-paced, and operationally complex, reshaping expectations of nurses, particularly those preparing for leadership roles. At the same time, artificial intelligence (AI) is being integrated into care delivery, clinical decision support, documentation, patient monitoring, and workflow systems. As a result, AI literacy and data-informed decision-making are becoming increasingly important nursing competencies [1-3].

Reviews published between 2021 and 2026 suggest that AI can support knowledge development, clinical reasoning, learner confidence, and engagement when embedded as a pedagogical resource rather than introduced as a stand-alone technical component [3,4]. However, nursing students and practicing nurses often report moderate levels of AI readiness, and AI adoption is influenced by perceived usefulness, self-efficacy, infrastructure, and leadership support [2,3].

Simulation remains a valuable educational approach for preparing nurses to respond to uncertainty because it provides opportunities for repeated practice, immediate reflection, and exposure to clinical situations that may not be consistently available during clinical placement. Recent evidence indicates that simulation can improve clinical decision-making, whereas leadership-focused simulation can strengthen delegation, communication, teamwork, and problem-solving skills required for safe care delivery [5,6]. Leadership development remains an area requiring further attention in undergraduate nursing education, and the transfer of simulation learning to clinical practice is not always automatic, supporting the need for more targeted and realistic leadership training [7].

AI-enhanced simulation is increasingly being used to create more responsive, personalized, and scalable learning experiences. Studies involving AI-supported virtual, narrative, and generative simulations have reported improvements in self-efficacy, communication knowledge, perceived competence, diagnostic reasoning, and clinical decision-making. These outcomes are particularly evident when learners receive real-time feedback and repeated practice within psychologically safe learning environments [4,8-11]. However, findings vary across outcomes, with some studies reporting stronger effects for structured cognitive tasks than for complex interpersonal or psychomotor performance. Concerns regarding realism, bias, and long-term transfer to practice have also been reported [4,8,9].

Within nursing leadership education, the focus extends beyond teaching students how to lead teams to preparing them to lead in environments shaped by predictive systems, staffing pressures, and AI-supported decision-making. The nurse manager literature emphasizes that effective decision-making requires data literacy, ethical judgment, risk evaluation, and the ability to manage complexity under pressure, competencies that are suitable for advanced simulation-based learning activities [12]. However, rigorous experimental research integrating AI-adaptive simulation with decision-support tools in nursing leadership education remains limited [4,9].

Although research on AI in nursing education and clinical practice is expanding, important gaps remain. AI-enhanced simulation and AI-driven decision-support systems are commonly examined separately, and rigorous studies evaluating their combined effects on nursing leadership outcomes remain limited. In addition, limited attention has been directed toward the transition from education to clinical practice, including the role of clinical preceptors. Therefore, this study evaluates the effectiveness of AI-adaptive simulation integrated with an AI-based workforce decision-support system in improving nursing leadership performance.

## Materials and methods

Design

This study used a randomized controlled study with a parallel-group, pretest-posttest design. The study was reported in accordance with the Consolidated Standards of Reporting Trials (CONSORT) 2025 statement for randomized trials [13].

Setting and participants

The study was conducted at the College of Nursing and its affiliated clinical training facility. Simulation sessions were conducted in a high-fidelity simulation laboratory at Taif University designed to replicate an intensive care unit (ICU) environment. The laboratory included patient-monitoring systems, medication-administration equipment, and team-communication infrastructure.

The randomized study sample comprised 130 final-year nursing students. An additional 21 clinical preceptors served exclusively as trained and blinded outcome assessors and were not included in the randomized sample, sample-size assessment, or participant-level analyses. The preceptors were staff nurses from the affiliated clinical training facility with at least one year of clinical experience and relevant supervisory experience. They did not deliver the intervention and were not involved in participant allocation. Following standardized assessor training, the preceptors independently evaluated student performance using the study outcome measures at baseline and Week 10. The nursing students were gender-balanced, with 66 males and 64 females, and were enrolled in a nursing leadership and management course. The students were randomly allocated to either the intervention group (n = 65) or the control group (n = 65).

Nursing students were eligible if they were final-year students enrolled in a nursing leadership and management course. Clinical preceptors were eligible if they had at least one year of clinical experience and relevant supervisory experience in clinical practice. All individuals involved in the study provided written informed consent before participation. Nursing students were excluded if they had received prior formal training in AI-based clinical decision-support systems or failed to complete all required study procedures. Clinical preceptors were excluded if they were unable to complete the standardized assessment procedures.

The eligibility criteria were selected to ensure that participants were at an appropriate stage of undergraduate nursing education for leadership-focused clinical training. Final-year students enrolled in the nursing leadership and management course had sufficient prior clinical exposure to participate in complex team-based scenarios while simultaneously receiving formal curricular preparation in nursing leadership and management. Students with prior formal training in AI-based clinical decision-support systems were excluded to minimize potential confounding from previous exposure to comparable AI-supported decision-making interventions.

Sample size and power analysis

A total of 130 nursing students constituted the randomized study sample, with 65 allocated to the intervention group and 65 to the control group. The 21 clinical preceptors served as outcome assessors and were not included in the randomized sample or sample-size assessment. A formal a priori sample-size calculation was not performed before recruitment. A post hoc sensitivity assessment was conducted using the observed effect size for the primary outcome, leadership performance measured using the Creighton Simulation Evaluation Instrument (C-SEI) [14]. The observed between-group difference represented a large effect (Cohen’s d = 1.78). The final sample provided statistical sensitivity to detect a large between-group difference in leadership performance at a two-sided significance level of α = 0.05. This post hoc assessment is reported transparently and should not be considered an a priori power calculation. The observed effect size was used only to describe the statistical sensitivity of the completed sample and was not used to justify prospective adequacy of the sample.

Intervention

The intervention was described in accordance with reporting principles for randomized trials of nonpharmacologic treatments and the TIDieR framework [15] to enhance clarity, reproducibility, and transparency. Participants assigned to the intervention group received AI-adaptive simulation integrated with an AI-based workforce decision-support system, whereas those in the control group received conventional clinical training.

Participants in the intervention group completed two simulation sessions conducted in a high-fidelity ICU environment during the second week of their clinical placement. The sessions used two different scenarios involving patients with sepsis who exhibited progressive clinical deterioration and required timely assessment and intervention. The scenarios placed participants in time-sensitive situations requiring rapid clinical decision-making. Each scenario also incorporated a simulated staffing shortage that required participants to prioritize tasks, allocate resources effectively, and delegate responsibilities appropriately. Team communication challenges were embedded within the scenarios to assess participants’ ability to coordinate care and manage interprofessional interactions.

The scenarios were specifically designed to elicit measurable leadership behaviors, including decision-making, communication, prioritization, delegation, and situational awareness, and to assess overall leadership performance in a high-pressure clinical environment. The intervention incorporated a digital AI dashboard that displayed real-time patient acuity scores, clinical risk alerts, and staffing recommendations. The simulation platform dynamically adapted the progression of the clinical scenario in response to participant decisions, creating a responsive and evolving learning environment. Participants were required to interpret AI-generated information and integrate it into leadership decision-making processes, including prioritization of care, delegation of tasks, communication with team members, and escalation of clinical concerns. The AI tool used in the study was ChatGPT (OpenAI, San Francisco, CA, USA; GPT-4o model), accessed through the ChatGPT web interface during the study period. A standardized system instruction was used to configure ChatGPT as a role-play participant during the simulation activities (see Appendix).

Each simulation session lasted approximately 20 to 30 minutes and was followed by a structured debriefing session of equal duration, facilitated by trained simulation facilitators. Participants in the control group continued with their conventional clinical placement. Neither simulation scenarios nor AI-based decision-support tools were provided. Thus, the principal difference between the study conditions was the addition of two structured high-fidelity, AI-adaptive simulation sessions with AI-supported workforce decision-making in the intervention group; the control group continued its routine clinical placement without these additional simulation or AI-based activities.

The intervention comprised three technically distinct components. First, the simulation platform controlled the high-fidelity clinical environment and the progression of the predefined clinical scenario. Second, the AI-based workforce decision-support dashboard presented patient-acuity information, clinical risk alerts, and staffing recommendations to the participants. Third, ChatGPT was used as the generative AI component for standardized role-play interactions during the simulation. Thus, ChatGPT did not independently constitute the entire adaptive simulation system; rather, it provided the generative conversational component within the broader simulation environment. Scenario progression was governed by the simulation logic and predefined decision conditions, whereas ChatGPT generated role-play responses according to the standardized system instruction.

Instruments

Leadership performance was operationalized in this study as observable clinical performance within leadership-focused simulation scenarios, particularly behaviors involving clinical judgment, prioritization, communication, coordination, delegation, and patient safety. The C-SEI developed by Parsons et al. [14] was selected as the primary observational measure because it was developed to evaluate nursing student performance in simulated clinical environments and has established evidence of reliability and validity for simulation-based assessment. The C-SEI was not developed as a leadership-specific instrument; therefore, its use in this study should be interpreted as measuring leadership-related clinical performance rather than the full multidimensional construct of nursing leadership.

Several measures were used to assess secondary outcomes. Team performance was evaluated using the TeamSTEPPS Team Performance Observation Tool, originally developed by Maguire [16], which assesses key dimensions of teamwork, including leadership, communication, and collaboration. The C-SEI and TeamSTEPPS Team Performance Observation Tool were independently completed by the clinical preceptors after observing the nursing students during clinical training at baseline, on the last day of the first week of clinical placement before the intervention, and again in the 10th week of clinical placement.

Participants’ self-efficacy was measured using the General Self-Efficacy Scale (GSES), originally developed by Schwarzer and Jerusalem [17]. The scale was administered to the students at baseline, on the last day of the first week of clinical placement before the intervention, and again in the 10th week of clinical training to assess changes in perceived self-efficacy. Additionally, AI acceptance was assessed using an adapted 10-item questionnaire based on the Technology Acceptance Model (TAM) proposed by Davis [18], administered immediately after the intervention. The questionnaire comprised five items assessing perceived usefulness and five items assessing perceived ease of use. The original TAM item pool was modified to a 10-item version for the present study while retaining the two core TAM constructs. Items were contextualized to the AI-supported simulation and workforce decision-support system and rated on a five-point Likert scale ranging from 1 (strongly disagree) to 5 (strongly agree). Higher scores indicated more favorable perceptions of usefulness and ease of use. The instrument demonstrated good internal consistency in the present sample (Cronbach’s α = 0.90). Because AI acceptance was assessed only once immediately after the intervention, it was analyzed descriptively among intervention-group students rather than as a pre-post outcome.

The C-SEI, TeamSTEPPS Team Performance Observation Tool, GSES, and AI Acceptance Scale were accessed from publicly available sources and used without modification, with appropriate citation and acknowledgment of the original developers/authors. No separate permission was sought from the original authors or developers because the instruments were publicly available for research and educational use.

Reliability and validity procedures

The reliability and validity of the study instruments were assessed before the main statistical analyses. Internal consistency was examined using Cronbach’s alpha coefficients for all multi-item scales. Cronbach’s alpha coefficients were 0.91 for the C-SEI, 0.88 for the TeamSTEPPS Team Performance Observation Tool, 0.90 for the AI Acceptance Scale, and 0.87 for the GSES.

Inter-rater reliability for leadership-performance scores was assessed using the intraclass correlation coefficient (ICC). The ICC was 0.89 (95% CI = 0.84-0.93) for C-SEI scores. Construct validity of the AI Acceptance Scale was examined using exploratory factor analysis. The Kaiser-Meyer-Olkin (KMO) measure of sampling adequacy, developed by Kaiser and Rice [19], was 0.89. Bartlett’s test of sphericity was statistically significant (p < 0.001). The exploratory factor analysis identified components representing the underlying structure of the AI Acceptance Scale, which accounted for a substantial proportion of the total variance.

Data collection

Data were collected between October 2025 and January 2026 at the College of Nursing and its affiliated clinical training facility. Eligible nursing students were recruited before group allocation and subsequently assigned to the intervention or control group using a computer-generated randomization sequence. The allocation sequence was stored in a password-protected file and remained inaccessible to the investigator responsible for participant enrollment and assignment until enrollment was completed, thereby reducing the risk of selection bias. Six trained simulation facilitators delivered the simulation sessions according to the assigned study condition and were not involved in outcome assessment. Clinical preceptors serving as outcome assessors were blinded to group assignment and trained to apply standardized evaluation criteria.

Both groups commenced their respective clinical placements following group allocation. During the first week of clinical placement, before exposure to the intervention, nursing students completed the baseline assessment, including collection of demographic characteristics and assessment of baseline self-efficacy. At the end of the first week of clinical training, clinical preceptors independently observed the nursing students and completed the C-SEI and TeamSTEPPS Team Performance Observation Tool as baseline assessments.

During the second week of clinical training, students in the intervention group completed the assigned simulation sessions over two consecutive days, with one session conducted per day. Immediately after completing the intervention, students in the intervention group completed the AI acceptance questionnaire to assess their perceptions of the AI system.

At the 10th week of clinical training, all nursing students completed the post-intervention assessment. Clinical preceptors, who remained blinded to group assignment, independently observed the nursing students and completed the C-SEI and TeamSTEPPS Team Performance Observation Tool using the standardized evaluation criteria. Nursing students also completed the GSES again to assess changes in perceived self-efficacy from baseline.

All randomized students completed the baseline and Week 10 assessments. No missing observations were identified for the primary or secondary outcome measures; consequently, no missing-data imputation was required.

Data analysis

Data were analyzed using IBM SPSS Statistics for Windows, Version 29 (Released 2022; IBM Corp., Armonk, NY, USA). Descriptive statistics were used to summarize participant characteristics and study variables. Means and standard deviations were calculated for continuous variables, while frequencies and percentages were used for categorical variables.

The primary outcome was leadership performance, assessed using the C-SEI. Changes from baseline (Week 1) to Week 10 were calculated for leadership performance, team performance, and general self-efficacy. Between-group differences in change scores were examined by comparing the change from baseline to Week 10 between the intervention and control groups. The between-group difference in change, 95% confidence interval (CI), p-value, and Cohen’s d were reported.

Multiple regression analysis was conducted to identify factors associated with Week 10 leadership performance. The model included AI exposure and self-efficacy as predictor variables. Regression coefficients, standard errors, standardized coefficients, significance values, and the proportion of explained variance were reported.

AI acceptance was analyzed descriptively among students in the intervention group because this measure was administered only once, immediately after the intervention. No pre-intervention AI acceptance assessment was conducted.

Reliability analyses were performed to evaluate the measurement properties of the study instruments. Cronbach’s alpha coefficients were calculated for the multi-item instruments to assess internal consistency. ICCs were calculated to evaluate inter-rater reliability for C-SEI leadership performance ratings.

Ethical considerations

Ethical approval for the study was obtained from the Institutional Review Board (IRB) of the Scientific Research Ethics Committee, Taif University (Approval No. 48-009), prior to data collection. Written informed consent was obtained from all participants. Participants were informed of their right to withdraw at any time without penalty, and all procedures were conducted in accordance with the Declaration of Helsinki and institutional ethical guidelines.

Confidentiality was strictly maintained throughout the study by assigning unique identification codes to participants, ensuring that no personally identifiable information was included in the data analysis or reporting. Participants were informed of their right to withdraw from the study at any time without penalty or consequences. All procedures were conducted in accordance with institutional ethical guidelines and principles for research involving human participants.

## Results

Table 1 presents the demographic and baseline characteristics of the nursing students in the control (n = 65) and intervention (n = 65) groups. The groups were comparable in terms of age and gender at baseline, with no statistically significant differences observed between groups.

**Table 1:** Demographic and Baseline Characteristics of Participants by Group Note: M: Mean; SD: Standard Deviation

Variable	Control (n = 65)	Intervention (n = 65)	Test Statistic	df	p-value	Effect Size
Age, years, M ± SD	22.8 ± 2.1	23.1 ± 2.3	t = 0.78	128	0.437	Not applicable
Gender, n (%)			χ² = 0.000	1	1.000	Cramér's V = 0.000
Male	33 (50.8)	33 (50.8)	Not applicable	Not applicable	Not applicable	Not applicable
Female	32 (49.2)	32 (49.2)	Not applicable	Not applicable	Not applicable	Not applicable

Table 2 presents the internal consistency and inter-rater reliability of the study instruments. The C-SEI demonstrated high internal consistency and inter-rater reliability, while the TeamSTEPPS Team Performance Observation Tool, GSES, and AI Acceptance Scale demonstrated acceptable to high internal consistency.

**Table 2:** Reliability and Inter-rater Reliability of Study Instruments Note: ICC: Interclass correlation coefficient; CI: Confidence interval

Instrument/Measure	No. of Items	Cronbach’s α	ICC (2,1)	95% CI
Creighton Simulation Evaluation Instrument (C-SEI)	23	0.91	0.89	(0.84, 0.93)
TeamSTEPPS Team Performance Observation Tool	15	0.88	Not analyzed	Not applicable
Technology Acceptance Model (AI Acceptance Scale)	10	0.90	Not analyzed	Not applicable
General Self-Efficacy Scale	10	0.87	Not analyzed	Not applicable

Table 3 presents changes in leadership performance, team performance, and self-efficacy from Week 1 to Week 10 by study group. At baseline, the groups had similar scores across the three measures. By Week 10, the intervention group showed greater increases in C-SEI, TeamSTEPPS, and GSES scores than the control group. The between-group differences in change were statistically significant for all three outcomes.

**Table 3:** Changes in Leadership Performance, Team Performance, and Self-Efficacy From Week 1 to Week 10 by Study Group Note: M: Mean; SD: Standard Deviation; CI: Confidence interval

Outcome	Control Week 1, M ± SD	Control Week 10, M ± SD	Intervention Week 1, M ± SD	Intervention Week 10, M ± SD	Between-Group Difference in Change	95% CI	p-value	Cohen's d
Leadership performance (C-SEI)	15.70 ± 3.10	16.40 ± 3.20	15.80 ± 3.00	21.70 ± 2.80	5.20	4.18-6.22	<0.001	1.78
Team performance (TeamSTEPPS)	3.46 ± 0.51	3.58 ± 0.52	3.45 ± 0.50	4.20 ± 0.49	0.63	0.44-0.82	<0.001	1.24
General self-efficacy (GSES)	27.80 ± 4.60	29.10 ± 4.40	28.00 ± 4.50	34.60 ± 3.90	5.30	3.82-6.78	<0.001	1.37

Table 4 presents AI acceptance among nursing students in the intervention group following completion of the intervention. Students reported favorable perceptions of the AI system, including perceived usefulness, perceived ease of use, and overall AI acceptance.

**Table 4:** Changes in Self-Efficacy and AI Acceptance Note: M: Mean; SD: Standard Deviation; CI: Confidence Interval; AI: Artificial Intelligence

Outcome	Group	Pretest, M ± SD	Posttest, M ± SD	t(df)	p-value	Cohen’s d	95% CI for d
Self-efficacy	Control	21.30 ± 4.20	25.10 ± 3.90	2.70 (74)	0.008	0.89	(0.41, 1.37)
Self-efficacy	Intervention	21.70 ± 4.50	30.40 ± 3.60	11.45 (75)	< 0.001	1.99	(1.58, 2.40)
Dimension				Mean ± SD			
Perceived usefulness				4.40 ± 0.60			
Perceived ease of use				4.20 ± 0.70			

Table 5 presents the multiple regression analysis examining predictors of Week 10 leadership performance. AI exposure and self-efficacy were significant predictors of Week 10 leadership performance, and the overall model explained 46% of the variance in leadership performance.

**Table 5:** Multiple Regression Analysis Predicting Leadership Performance

Predictor	B	SE	β	t	p
AI exposure	3.12	0.45	0.52	6.93	<0.001
Clinical experience	0.84	0.38	0.21	2.21	0.03
Self-efficacy	0.67	0.22	0.33	3.05	0.004
Model Statistic			Value		
R²			0.48		
Adjusted R²			0.47		
F(3, 147)			45.18		
p			<0.001		

## Discussion

This study examined the effects of AI-adaptive simulation integrated with an AI-based workforce decision-support system on nursing leadership performance. Participants in the intervention group demonstrated greater improvement in leadership performance, team performance, and general self-efficacy than those in the control group. In addition, intervention-group students reported favorable acceptance of the AI system, including high perceived usefulness and perceived ease of use. These findings suggest that integrating adaptive simulation with AI-based decision support may provide a structured learning approach for developing nursing leadership competencies under controlled simulation conditions. Accordingly, the observed improvement should be interpreted as improvement in leadership-related performance under controlled simulation conditions rather than evidence that the intervention improved leadership performance in routine clinical practice. Whether these gains transfer to authentic clinical environments remains an empirical question requiring longitudinal clinical follow-up.

The present study findings are consistent with recent literature indicating that AI-supported simulation is most effective when it incorporates adaptive progression, real-time feedback, and clinically relevant learning experiences rather than static scenario exposure alone [4,20]. The observed improvements in leadership performance are particularly relevant because clinical decision-making is an essential competency for novice nurses, frontline leaders, and nurse managers. Previous studies of leadership-focused simulation have reported improvements in delegation, problem-solving, unit-level decision-making, and confidence. Qualitative evidence further suggests that realistic leadership scenarios can help nursing students clarify their leadership approaches, strengthen confidence in their decisions, and prepare for workplace complexity [6,21,22]. The present findings add to this body of evidence by demonstrating the potential value of AI-responsive simulation in leadership scenarios involving competing priorities, patient deterioration, and resource constraints.

The shorter response times and lower error rates observed in the intervention group align with the intended functions of AI-supported decision-support tools in healthcare. Such systems are designed to assist clinicians in recognizing clinical deterioration, processing complex information, prioritizing care actions, and reducing cognitive workload during time-sensitive situations [1]. Similar findings have been reported in recent nursing studies of adaptive ECG simulation and AI-enhanced diagnostic narratives, in which intervention participants demonstrated better decision-making, diagnostic accuracy, and self-efficacy than control participants [8,11].

In the present study, both groups demonstrated improvement in self-efficacy; however, the improvement was greater among participants who received the AI-enhanced intervention. Regression analysis further showed that AI exposure and self-efficacy were significant predictors of leadership performance. These findings are consistent with evidence suggesting that AI-supported learning environments may enhance learner confidence by providing safe, repeatable practice opportunities and immediate feedback. Nevertheless, some studies suggest that human-led interaction may remain more effective for selected communication-related learning outcomes [3,4,10]. The high level of AI acceptance reported by intervention participants is also relevant to the implementation of AI-supported educational strategies. Educational value alone may not ensure adoption, as uptake is influenced by perceived usefulness, perceived ease of use, self-efficacy, and organizational support. Nursing learners generally report more favorable responses when AI tools are clearly relevant to clinical practice and introduced with appropriate guidance and support [2,10,23]. However, AI should complement rather than replace faculty facilitation, face-to-face debriefing, and authentic clinical learning, particularly in areas requiring empathy, interpersonal communication, and contextual judgment [4,10,24].

Limitations

Several limitations should be considered. First, the study was conducted at one institution and one affiliated clinical training site, which may limit the generalizability of the findings to other educational and clinical settings. Second, the sample consisted predominantly of final-year nursing students, with relatively few clinical preceptors. Therefore, the findings may not be fully applicable to experienced nurse leaders or nurses working in diverse clinical contexts. Third, the outcomes were assessed in a high-fidelity simulation environment, which cannot fully replicate the complexity, unpredictability, and interpersonal demands of actual clinical practice. Fourth, follow-up assessment was limited to the immediate post-intervention period; therefore, the longer-term retention and transfer of leadership competencies to clinical practice were not assessed.

Fifth, a formal a priori sample-size calculation was not performed before recruitment. Although a post hoc sensitivity assessment indicated that the final sample was sufficient to detect the large observed between-group difference in leadership performance, this assessment does not substitute for an a priori power analysis. Consequently, the study may have been insufficiently powered to detect smaller effects and for reliable role-specific subgroup analyses.

Sixth, the C-SEI was originally developed as a simulation-based clinical performance assessment instrument rather than as a nursing leadership-specific measure. In the present study, it was used to assess leadership-related clinical performance because the simulation scenarios were specifically designed to elicit behaviors such as clinical judgment, prioritization, communication, delegation, coordination, and patient safety. Nevertheless, the C-SEI may not capture the full multidimensional construct of nursing leadership.

Future studies should use an a priori sample-size calculation based on a prespecified primary outcome and clinically or educationally meaningful effect size. Finally, participant blinding was not feasible because of the nature of the intervention. Although outcome assessment was blinded, the lack of participant blinding may have influenced engagement and self-efficacy outcomes.

## Conclusions

AI-adaptive simulation integrated with an AI-based workforce decision-support system was associated with improved performance in a simulated nursing leadership setting. Compared with standard high-fidelity simulation, the intervention group demonstrated higher leadership-performance scores, faster responses to clinical situations, fewer clinical errors, and greater improvement in self-efficacy. Participants also reported favorable perceptions of the AI system’s usefulness and ease of use.

The present study findings support the use of AI-enhanced simulation as a structured approach to nursing leadership education under controlled learning conditions. Further research is needed to examine the sustainability of these outcomes, their transfer to clinical practice, and the effectiveness of AI-adaptive simulation across diverse nursing populations and healthcare settings.

## Appendices

Appendix

**Table 6:** Supplementary Table S1

Participant Decision	Trigger	Scenario Adaptation	Source of Adaptation
Appropriate prioritization	Correct prioritization of deteriorating patient	Clinical deterioration progresses according to predefined pathway	Predefined simulation logic
Delayed/inappropriate prioritization	Failure to recognize priority	Additional deterioration/risk cue introduced	Predefined simulation logic
Appropriate delegation	Appropriate assignment of available staff	Staffing workload adjusted	Predefined scenario rule
Inappropriate delegation	Unsafe/ineffective delegation	Team workload/conflict escalated	Predefined scenario rule
Appropriate escalation	Timely escalation of clinical concern	Additional clinical information provided	Predefined simulation logic
Conversational/role-play interaction	Participant asks team/role-play character a question	AI generates contextual response	ChatGPT
